# Practice facilitation and academic detailing improves colorectal cancer screening rates in safety net primary care clinics

**DOI:** 10.1186/1748-5908-10-S1-A57

**Published:** 2015-08-20

**Authors:** Emily M Mader, Chester H Fox, Karen Vitale, Angela M Wisniewski, John W Epling, Gary N Noronha, Carlos M Swanger, Amanda L Norton, Christopher P Morley

**Affiliations:** 1 Department of Family Medicine, State University of New York Upstate Medical University, Syracuse, NY, 13210, USA; 2 Department of Family Medicine, State University of New York at Buffalo, Buffalo, NY, 14221, USA; 3 Center for Community Health, University of Rochester School of Medicine and Dentistry, Rochester, NY, 14642, USA; 4 Center for Primary Care, University of Rochester School of Medicine and Dentistry, Rochester, NY, 14642, USA

## Objective

SUNY Upstate Medical University entered a contract with Health Research, Inc. and the New York State Department of Health to implement an intervention using academic detailing and practice facilitation to increase colorectal cancer screening rates within primary care practices, and to assess the outcomes and barriers to intervention success. The project was conducted within a large multi-organizational framework, led by the Studying-Acting-Learning-Teaching Network (SALT-Net, SUNY Upstate Medical University) in partnership with the Upstate New York Network (UNYNET -University at Buffalo) and the Greater Rochester PBRN (GR-PBRN -University of Rochester Medical Center), under the auspices of the Upstate New York Translational Research Network (UNYTE).

## Methods

Nine primary care practices enrolled in the project. Practices received a 1-hour academic detailing session on colorectal cancer screening guidelines, and two months of practice facilitation services to implement evidence based strategies to increase patient screening, with a particular focus on initiating and/or streamlining the use of electronic health record (EHR)-based patient screening registries. The impact of project participation on colorectal cancer screening as assessed through pre-and post-intervention screening rates.

## Results

Primary intervention activities completed by participating practices included efforts to address the data recording issues within practice EHR systems, provider feedback and assessment activities, streamlining of provider reminder systems, and patient education and outreach interventions. The difference between mean pre-and post-intervention screening rates was statistically significant (mean pre-rate 24.7% (SD 20.6%) vs. mean post-rate 26.4% (SD 20.5%), p = 0.028). A preliminary assessment indicates a mean relative increase of 30% between the pre-and post-screening rates.

## Relevance to dissemination and implementation

This project evaluates the efficacy of a targeted intervention to implement evidence-based practices in a primary care setting to increase colorectal cancer screening rates, and provides tangible information on facilitators and barriers to implementing these practices in safety net clinics.

